# The Origin of Vasomotion and Stochastic Resonance in Vasomotion

**DOI:** 10.3389/fbioe.2022.819716

**Published:** 2022-03-02

**Authors:** Shuhong Liu, Liangjing Zhao, Yang Liu

**Affiliations:** ^1^ Research Centre for Fluid-Structure Interactions, Department of Mechanical Engineering, The Hong Kong Polytechnic University, Hung Hom, Hong Kong SAR, China; ^2^ Department of Mechanical Engineering, Kowloon, Hong Kong SAR, China

**Keywords:** vasomotion, spectral analysis, simulation, stochastic resonance (SR) noise, origin of vasomotion

## Abstract

Vasomotion is the spontaneous time-dependent contraction and relaxation of micro arteries and the oscillating frequency is about 0.01–0.1 Hz. The physiological mechanism of vasomotion has not been thoroughly understood. From the dynamics point of view, the heartbeat is the only external loading exerted on the vascular system. We speculate that the nonlinear vascular system and the variable period of the heartbeat might induce the low-frequency vasomotion. In this study, the laser Doppler flowmeter is used to measure the time series of radial artery blood flow and reconstructed modified time series that has the same period as the measured time series but different heartbeat curves. We measured the time series of radial artery blood flow in different conditions by adding different noise disturbances on the forearm, and we decomposed the experiment pulse signal by Hilbert–Huang transform. The wavelet spectral analyses showed that the low-frequency components were induced by the variable period but independent of the shape of the heartbeat curve. Furthermore, we simulated the linear flow in a single pipe and the nonlinear flow in a piping network and found that the nonlinear flow would generate low-frequency components. From the results, we could deduce that the variable period of heartbeat and the nonlinearity of the vascular system induce vasomotion. The noise has effects on the blood signals related to the respiratory activities (∼0.3 Hz) but little influence on that related to the cardiac activities (∼1 Hz). Adding white noise and then stopping would induce an SNR increase in the frequency band related to vasomotion (∼0.1 Hz).

## 1 Introduction

Vasomotion is the spontaneous change in micro arteries diameters, which was first observed in the bat wing (Jones, 1852; Gustafsson et al., 1993; Rossi et al., 2006). Microcirculation may reflect the conditions of other systemic vascular functions and the microvascular abnormalities may originate the pathogenesis sequence in some diseases (Holowatz et al., 2008), and the link between vasomotion and cardiovascular diseases is acknowledged in many research studies (Fonseca et al., 2018; Smith, 2020). However, the mechanism responsible for vasomotion is still unknown. Many studies (Mück-Weymann et al., 1996; Bracic and Stefanovska, 1998; Stefanovska et al., 1999; Söderström et al., 2003; Cracowski et al., 2006) analyzed characteristic frequencies of blood flow oscillation signals, and these frequencies components correspond to several types of activities. The oscillations in the region around ∼1 Hz are related to the cardiac activity, the oscillations in the region around ∼0.3 Hz are related to the respiratory activity, and the oscillations ranging from 0.001 to 0.2 Hz are related to endothelial and myogenic activities (Stefanovska et al., 1999; Söderström et al., 2003). Vasomotion is the oscillations related to endothelial and myogenic activities whose frequency interval is from 0.001 to 0.2 Hz. Recently, researchers have investigated the role of vasomotion in diabetes. Experimental results show significant differences in vasomotion between diabetic patients, prediabetic patients, and nondiabetic people (Hsiu et al., 2018). Low-frequency pulsations of the blood flow associated with endothelial activities are lower in diabetic patients (Mizeva et al., 2018). Diabetic patients were found to have endothelial dysfunction in some arteries (De Ciuceis, 2020). Detecting microvascular dysfunction before diabetic complications could play an important role in the pre-diagnosis of diabetes (Fredriksson et al., 2021). There is physiological significance to provide insight into different microvascular impacts. The method of Laser-Doppler flow (LDF) signal is applied in extensive studies of human skin circulation in the last half-century because the LDF signal achieves an appraisal of tissue blood flow (Schmidt et al., 1993). LDF uses the Doppler effect in the illuminated tissue to calculate the blood flow (or flux) and can be used to detect the blood flow oscillations (Nilsson et al., 1980; Bollinger et al., 1991). After photons in light beams are shot into the tissue, Doppler shifts induced by moving erythrocytes are evaluated by the cell’s scattering angle, wavelength, and velocity vector (Rajan et al., 2009). LDF is completely non-invasive and real-time at high sampling rates, and the frequency features of LDF signals can be processed with wavelet analysis (Schabauer and Rooke, 1994).

*In vivo*, vasomotion may be influenced by various mechanical stresses exerted on the smooth muscle cells and endothelial cells. Blood pressure generates circumferential stress in smooth muscle cells and endothelial cells, and endothelial cells are also directly subject to fluid shear stress. It has been shown that intraluminal pressure changes may induce vasoconstriction, a phenomenon known as the myogenic response (Koenigsberger et al., 2006). Meanwhile, vasomotion is related to the intracellular oscillatory processes in smooth muscle cells. A cytosolic oscillator, a membrane oscillator, and a metabolic oscillator lead to rhythmical contraction and relaxation of vascular smooth muscle (Siegel, 1983; Lamb et al., 1985; Bollinger et al., 1993; Gustafsson et al., 1993). Some experimental studies found that vasomotion is achieved through electrical communication and depends on Ca^2+^-activated K^+^ channels in endothelial cells (Mauban and Wier, 2004). The acoustic shear wave, as mechanical energy, is able to cause electrochemical activity. In Li et al.’s experiments (Li et al., 2011), a needle’s motion generated acoustic shear waves to initiate cytosolic Ca^2+^ rise in both excitatory and non-excitatory cells, to induce Ca^2+^ oscillations, and to augment *in vivo* calcium liberation into blood plasma of mice. On the other hand, the periodic transports of Ca^2+^ and K^+^ flux introduce a time-periodic cytoplasmic calcium concentration, the cross-bridges formation in smooth muscles, and the development of muscle stress. The resultant muscle stresses determine the rate of change of the vessel’s diameter: vasomotion (Lamb et al., 1985; Koenigsberger et al., 2006). Therefore, a possible pathway is that the blood flow generates wall shear stress which could induce vasomotion.

Traditionally the heartbeat is considered as a regular rhythm with a constant cardiac cycle. The heart rate, the number of heart beats per minute, is a nonstationary signal. The oscillations of a healthy heart reflect many physiological conditions adjusting the normal heart rhythm. (Shaffer and Ginsberg, 2017). Heart rate variation may indicate current disease or reveal potential cardiovascular risks (Rajendra Acharya et al., 2006). It has been noted that the cardiovascular system is not a simple linear system and involves nonlinear contributions (Rajendra Acharya et al., 2006). Moreover, it was found that the oscillating blood flow in microvascular networks exhibited nonlinear behavior (Carr and Lacoin, 2000). Heart rate variability (HRV) is alterations in the time intervals between adjacent heartbeats (Shaffer et al., 2014). From the dynamics point of view, the heartbeat is the only external excitation to the vascular system. Because the vascular network is a nonlinear system and the subharmonics would appear in a nonlinear system (Hayashi, 1953), the variable period of the heartbeat may induce the subharmonics in the vascular network. We speculate that the oscillation of blood flow generates shear stress which would develop muscle stress, and the variation of muscle stress induces vasomotion. If so, the variable period of heartbeat should induce the low-frequency component and is independent of the heartbeat curve. The determination of primary variables of fluid flux and average pressure in each cross-section of vascular could predict peak pressures (Daversin-Catty et al., 2021). In this paper we reconstructed the pulse signal with sinusoidal curves. A signal representing fluid flux in each heartbeat period is extracted from the reconstructed signal to investigate the frequency relationship between fluid flux and vasomotion. Also, we carried out CFD simulations to study the effect of a variable period of time series on low frequency.

By adding random perturbations, the feeble information in nonlinear system signals is amplified, discriminated, and optimized; this phenomenon refers to stochastic resonance (SR) which can be described by a response of the system to varying noise (Wellens et al., 2003). SR is discernible in nonlinear systems when the signal to noise ratio (SNR) ascends sharply to a peak value then decreases moderately due to a continuous increase of input noise intensity (Harmer et al., 2002). The three basic components of SR are an energetic activation barrier in the form of a threshold, a weak coherent input such as a periodic signal, and a noise source inherent in the system or added to the coherent input (Gammaitoni et al., 1998). The SR phenomenon is also observed in the “baroreflex” system and is involved in human blood pressure control (Shaffer et al., 2014). In the baroreflex system, baroreceptors detect blood pressure fluctuation and modulate heart rate as well as vascular tone. In both artery and vein, there are receptors in the baroreflex system located in the neck region monitoring the blood pressure. The brain stem receives the afferent inputs from receptors and transmits efferent outputs through a common pathway into the peripheral organs. Without adding noise, this weak subthreshold signal neither results in appreciable blood pressure responses nor stimulates the baroreflex response. After noise adding to the receptors, together with the weak signal inputs, an enhanced output response is produced which was conveyed via a common pathway load into the heart system. The threshold-like behavior is related to SR whose nonlinearity initiates the intensified baroreflex response in brain stem.(Hidaka et al., 2000; Yamamoto et al., 2005) SR involved in this process acts on the higher level of the brain stem, demonstrating the functional benefits of adding noise to the brain which optimizes the response (Hänggi, 2002). However, there is inherent background noise in the human cardiovascular system (Yamamoto and Hughson, 1994), and even without additional noise, the weak fluctuations in venous blood pressure will not result in perceptible heart rate responses but conversely alter the baroreflex sensitivity and trigger the arterial baroreflex (Hidaka et al., 2000). Therefore, the characteristic of the transmission process between blood pressure and the outputs of efferent neural activities is impractically simple linear in baroreflex physiology evaluation (Kubota et al., 1992; Triedman and Saul, 1994) and might be influenced by the intrinsic background noise. Above all, several studies are still looking into how to utilize unpredictable fluctuations in biology (McDonnell and Abbott, 2009), and such comprehension might be significant for the development of biomedical studies.

## 2 Methods

The investigation has been approved by the local ethics committee and is performed in accordance with the Declaration of Helsinki, and the subjects are provided with written informed consent forms.

### 2.1 Wavelet Analysis

In the study of skin blood flow regulation mechanisms, Fourier spectrum analysis and wavelet analysis are employed as two basic methods of spectral analysis (Stefanovska et al., 1999; Shaffer et al., 2014). Fourier spectrum analysis conveys the overall amplitude of a signal at particular frequencies which can be utilized to describe the cyclic pulses in skin blood flow. Wavelet is a zero-mean function located in both time and frequency domain and wavelet transform of a time series build a three-dimensional construction based on the time-frequency plane (Torrence and Compo, 1998). There are two types of wavelet: transform the continuous wavelet transform (CWT) and discrete wavelet transform (DWT). The CWT contributes to discovering the major oscillatory modes of a signal and the changing of those modes in times. Thus, the skin blood flow signals are suggested to be analyzed using continuous wavelet transform due to their continuously varying features. The CWT is given by

$$g\left(s,t\right)=\frac{1}{\sqrt{s}}\overset{\infty }{\underset{-\infty }{\int }}\psi \left(\frac{u-t}{s}\right)g\left(u\right)du$$

where *s* is the multiplier factor, *t* is the signal location over time, and ψ is the wavelet function. The wavelet used in this paper is a Morlet wavelet due to its advantage to balance time and frequency positioning (Paul, 2017). The complex Morlet wavelet is defined as

$$\psi \left(u\right)=\frac{1}{\sqrt[{4}]{\pi }}e^{i2\pi f_{0}u}e^{-u^{2}/2}$$

### 2.2 Hilbert–Huang Transform

Generally, the Fourier spectra give relevant descriptions of linear and stationary signals, and the analysis results are only capable of indicating global properties. Thus, it is controversial to apply Fourier spectrum analysis to nonlinear and nonstationary signals. In a real situation, most processes are neither linear nor stationary. Hilbert–Huang transform (HHT) is recently proposed and designed by Huang et al. (Huang et al., 1996; Huang et al., 1998; Huang et al., 1999) and is potentially viable in processing and analyzing nonlinear and nonstationary signals. This method is an empirical method that consists of empirical mode decomposition (EMD) and Hilbert spectral analysis (HSA) (Huang, 2014a). As a part of HHT, HSA is a way to present nonstationarity through obtaining instantaneous frequency and amplitude. The Hilbert transform y(t) for any real value signal x(t) is

$$y\left(t\right)=H\left[x\left(t\right)\right]=\frac{1}{\pi }PV\overset{\infty }{\underset{-\infty }{\int }}\frac{x\left(\tau \right)}{t-\tau }d\tau$$

where PV indicates the singular integral’s Cauchy principal value. By adopting the instantaneous amplitude $a\left(t\right)=\sqrt{x^{2}+y^{2}}$ , the instantaneous phase function $\theta \left(t\right)=\text{arctan}\left(\frac{y}{x}\right)$ , and the instantaneous frequency $\omega =\frac{d\theta }{dt}$ , the analytic signal can be defined as $z\left(t\right)=x\left(t\right)+iy\left(t\right)=a\left(t\right)e^{i\theta \left(t\right)}$ .

As a crucial part of HHT, the EMD is derived from the local signatures of nonlinearity. It is assumed that there are a finite number of oscillatory modes coexisting in the data, and these oscillatory modes have clearly different frequencies and are superimposed on each other at any particular time. Based on these assumptions, the EMD can decompose complex signals into intrinsic mode functions (IMFs). IMFs are defined with two requirements that (1) the difference between the number of extrema and the number of zero-crossings in the whole dataset must be zero or one and (2) at any point, the mean value of the envelope defined by the local maxima and the envelope defined by the local minima is zero (Huang and Wu, 2008). The IMF is extracted by the following steps:

1) Identifying all the local extrema of an input signal  x(t)

2) Forming the upper envelop and lower envelop by connecting all maxima and minima, respectively, and calculating their mean $m_{1}$

3) The difference $d_{1}$ between input data x(t) and their mean $m_{1}$ is calculated that $d_{1}=x\left(t\right)-m_{1}$ , and $d_{1}$ satisfies the definition of an IMF
4) Repeating the shifting process above after k times of iterations, $d_{1k}=d_{1\left(k-1\right)}-m_{1k}$ , and the IMF $c_{1}=d_{1k}$

5) The residual $r_{1}=x\left(t\right)-c_{1}$ is treated to be a new set of data and repeat the same shifting process $r_{n}=r_{n-1}-c_{n}$

6) Summing up the residual equations, $x\left(t\right)=\overset{n}{\underset{j=1}{\sum }}c_{j}+r_{n}$ , i.e., decomposing the input signal x(t) into IMFs $c_{n}$ and residual $r_{n}$

After the application of the Hilbert transform on each IMF component, the signal x(t) can be represented as

In studies (Huang and Attoh-Okine, 2005; Huang, 2014b), HHT is validated empirically and able to reveal signals’ true physical meanings in the time and frequency doma

$$x\left(t\right)=Re\left[\sum_{j=1}^{n}a_{j}\left(t\right)e^{i{\int \omega }_{j}\left(t\right)dt}\right]$$

in. This methodology provides much more apparent results than analysis results of traditional methods, particularly in time-frequency-energy representations (Huang, 2014a). Recently, HHT is applied to biomedical signal analysis, such as decomposes of electrocardiographic signal (Huang et al., 2008; Huang et al., 2009; Lin and Zhu, 2012; Fu et al., 2014) and ballistocardiograph signal (Stork et al., 2006; Stork and Trefny, 2010). By decomposing signals and extracting ambiguous information hidden behind the signals, the effects of external stimuli on the cardiovascular system may be determined.

The laser Doppler flowmeter was used to measure the time series of radial artery blood flow in different conditions by adding the white noise and pink noise on the forearm. After using Hilbert–Huang transform and wavelet analysis, we got instantaneous SNR and instantaneous noise of the blood flow signal and observed SR phenomenon in vasomotion.

### 2.3 Noise Type

White noise is a noise whose frequencies are distributed equally over a wide range of frequencies with uniform intensity. Colored noise is noise with uneven distribution of frequency components. Pink noise is one of the colored noises whose intensity is inversely proportional to the frequency, so all octaves have an equal amount of energy. In terms of power at a constant bandwidth, the pink noise decreases at a rate of 3 dB per octave.

### 2.4 Numerical Procedure

**FIGURE 1:**
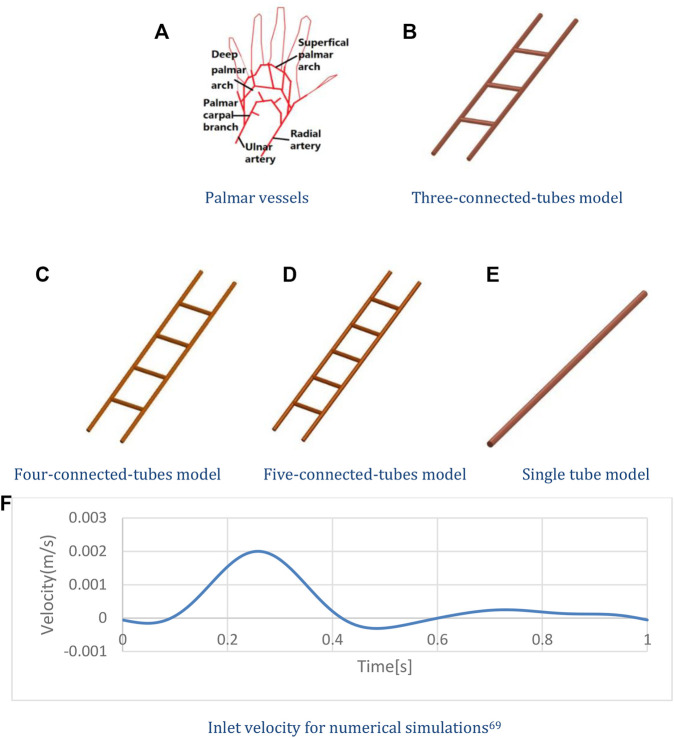
Numerical models and Inlet velocity. **(A)** Palmar vessels. **(B)** Three-connected-tubes model. **(C)** Four-connected-tubes model. **(D)** Five-connected-tubes model. **(E)** Single tube model. **(F)** Inlet velocity for numerical simulations.

The blood vessel model of palmar vessels is shown in Figure 1A. The three-connected-tubes vessel model shown in Figure 1B contains five tubes simplified from palmar arteries. Models with tubes of 1, 2, and 4 mm diameter are investigated separately. The length of the two main tubes is 100 mm, and the length of the connected tubes is 20 mm. The connected tubes are spaced at 25 mm intervals. The four-connected-tubes vessel model is shown in Figure 1C with connected tubes spaced at 20 mm intervals. The five-connected-tubes vessel model is shown in Figure 1D, and the connected tubes are located at the 17 mm, 33.5 mm, 50 mm, 66.5 mm, and 83 mm points on the main tubes. The diameter of the tubes in the four-connected-tubes vessel model and the five-connected-tubes vessel model are 2 mm. The nonlinear effect of the vascular system and its relationship to the low frequencies were investigated in a three-connected-tubes model using ANSYS CFX. The single tube model, as shown in Figure 1E, has a diameter of 2 mm and a length of 100 mm, and was studied as a linear system for comparison. The density of the blood was set as 1,050 kg/m^3^, and non-Newtonian properties were determined by the Casson model. The no-slip wall boundary condition was adopted to the vessel wall. The actual velocity waveform (Mills et al., 1970) shown in Figure 1F was adopted to simulate the inlet velocity in the radial artery.

### 2.5 Experiment Procedure

#### 2.5.1 The Origin of Vasomotion

We measured the oscillating radial artery blood flow at the wrist of four health subjects, two females and two males of ages from 20 to 30. The baseline characteristics for four subjects are presented in Table 1. They were asked (1) to avoid caffeine 2 hours before the test; (2) to remain seated and still during the measurement; and (3) to avoid drugs 3 days before the test.

**TABLE 1:** Anthropometric parameters for four subjects.

	Gender	Hight (cm)	Weight (kg)	Systolic Pressure (mmHg)	Diastolic Pressure (mmHg)	Pulse rate (BPM)
Subject 1	Female	150	50	67	108	73
Subject 2	Female	164	52	70	116	87
Subject 3	Male	168	55	73	115	78
Subject 4	Male	178	85	79	125	76

During measurement, two laser probes were connected to the Laser Doppler perfusion and the temperature monitor. Each probe contains one transmitting optical fiber and one receiving optical fiber. When attached to a specific skin location, probes will receive the Doppler-shifted laser light reflecting the activities of red blood cells which are traveling through vessels. A 40 Hz sampling rate, meaning a 0.025 s time interval, was used. The measurement time was 10 min.

**FIGURE 2:**
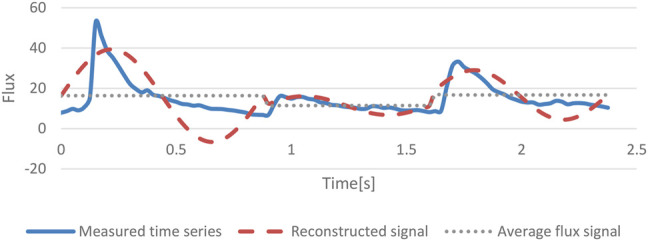
Reconstructed signal and measured time series in three cycles.

The measured time series exhibited a periodical pattern so that the whole measuring duration could be divided into beat-to-beat periods. Based on the heartbeat curve, we reconstructed two modified time series, the average flux signal and the reconstructed signal, period by period. The average flux curve is generated by averaging all the data points in each period. The reconstructed signal consists of sinusoidal curves and each sinusoidal curve is constructed by a sinusoidal function and a lift. The sinusoidal function’s amplitude A equals half of the difference between the maximum value $D_{max}$ and minimum value $D_{min}$ in each period. The lift is the average flux B in each period. After the reconstruction of the average flux curve and the sinusoidal curve, the irregular fluctuation on the heartbeat curve is eliminated and its influences on low frequencies are minimized. Figure 2 indicates the reconstructed time series and the measured time series in three periods with the sequence axis as the x-axis.

Reconstructed signal in each period=ASin(2πt/T)+B

$$\text{B}=\frac{\sum_{i=1}^{n}D_{i}}{\text{n}\;}$$

$$\text{A}=\;\frac{D_{max}-D_{min}}{2}$$

n—Number of measured data in each period.

${\text{D}}_{\text{i}}$ – Measured data in each period.

#### 2.5.2 Stochastic Resonance

**FIGURE 3:**
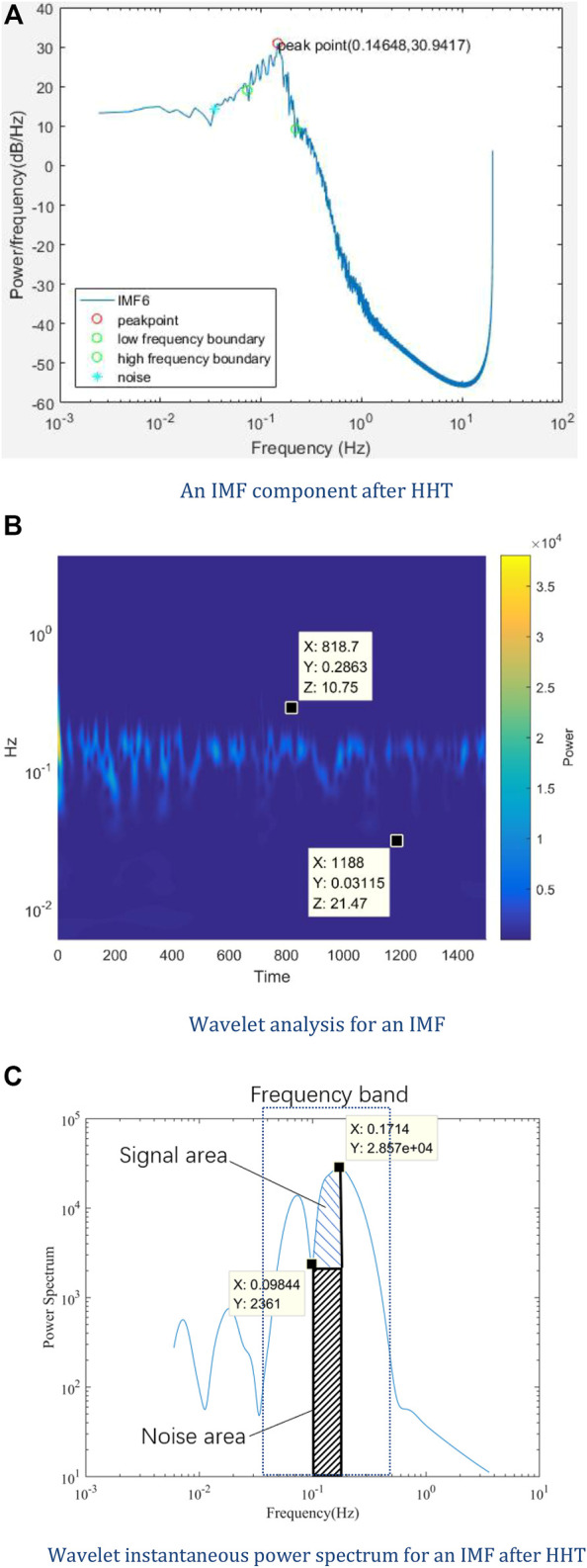
Data process procedure. **(A)** An IMF component after HHT, **(B)** Wavelet analysis for an IMF, **(C)** Wavelet instantaneous power spectrum for an IMF after HHT.

We measured the oscillating radial artery blood flow at the wrist for one health subject, ages 28. The sampling rate is 20 Hz which indicates that the time interval is 0.05 s. A speaker was placed on the forearm, and white noise (YouTube, 2021a) and pink noise (YouTube, 2021b) downloaded from YouTube were added to the pulse signal. All the subjects adopted a seated position during the measurement. The measurement time lasted 1 hour and 40 min, 20 min for each of the five periods: without adding noise, adding white noise, after adding white noise, adding pink noise, and after adding pink noise. HHT analysis of LDF signals was conducted. The signals were decomposed into several IMFs, one of which is shown in Figure 3A. Wavelet analysis was then applied to these IMFs, and a frequency band with an upper-frequency limit and a lower-frequency limit was set for each IMFs, which is indicated in Figure 3B. Through wavelet analysis, we could get the wavelet instantaneous power spectrum for IMFs after HHT. As shown in Figure 3C, because the right-hand-side spectrum of IMFs might be a declining slope, it is hard to determine the signal power and noise power of the right-hand-side half spectrum. Therefore, the SNR was calculated by the left-hand-side half of the spectrum curve. In the frequency band, all the extrema were identified, and the maxima and the minima power point were found among these extrema. Figure 3C shows the integrated signal power $S_{p}$ and the integrated noise power $N_{p}$ in the enclosed area of the spectrum curve under blues shades and black shades, respectively, both of which started from the minima power point and ended in the maxima power point.

## 3 Results

### 3.1 The Origin of Vasomotion

#### 3.1.1 Experiment

**FIGURE 4:**
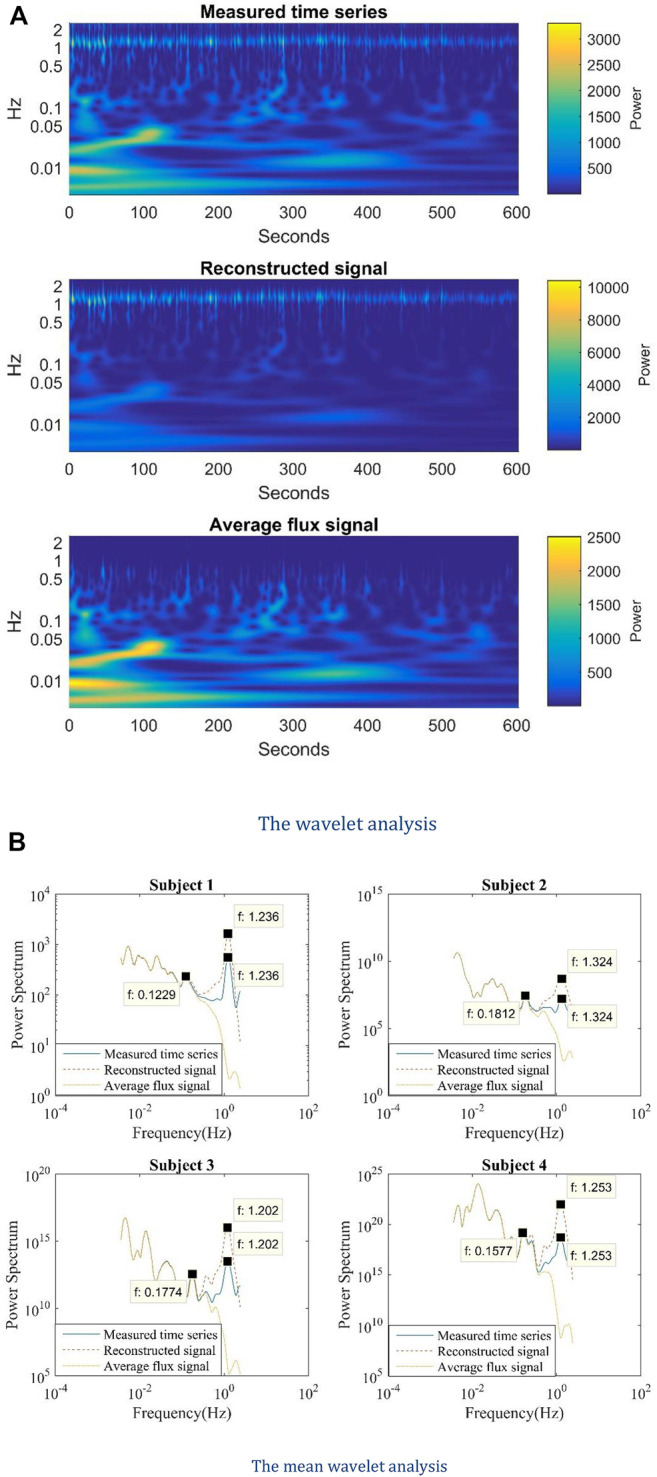
**(A)** The wavelet analysis and **(B)** the mean wavelet analysis of the measured time series, the reconstructed signal, and the average flux signal.

Figure 4A shows the wavelet analysis of the measured time series, reconstructed with sinusoid curve and average flux signal. In general, the frequency distributions of these three cases are similar to each other. The lower frequencies band is almost the same. In the case of the average curve, the frequency is very weak at 1 Hz but quite strong at the lower frequency band. To show the comparison clearly, we took the mean value along the time span, as shown in Figure 4B.

Figure 4B demonstrates the mean wavelet analysis of the measured time series, the reconstructed signal, and the average flux signal for the four study subjects. The measured and reconstructed sinusoid time series have the same heart rate peaks. The average flux signal can be extracted from either the measured time series or the reconstructed signal. So, the measured time series and the reconstructed signal contain all the information of the averaged flux data and share some oscillation features as the average flux signal. It is obvious in Figure 4B that the frequencies below 0.2 Hz are almost identical in the mean wavelet curves of these three signals. Thus, the shared lower frequency band (0.01–0.2 Hz) in the mean wavelet analysis results of the reconstructed signal and measured time series comes from the average flux. Because the average flux in each period represents the local average blood flux, it can be speculated that the lower frequency band (0.01–0.2 Hz) is induced by the averaged blood flux in each heart period.

Linear regression analysis helps to find the relationship between measured time series and reconstructed with sinusoid curves, and that between measured time series and average flux signals. The coefficients of determination (R^2^) interpret the ability of a model to predict or explain an outcome. In Table 2, the R^2^ of mean wavelet analysis data between reconstructed signals and measured time series, and that between average flux signals and measured time series, are all close to one for the four subjects. The average flux signals fit the measured time series better than reconstructed signals on account of larger R (Jones, 1852). Also, the intercepted mean wavelet analysis data of frequency below 0.25 Hz generates an even larger R (Jones, 1852). The lower frequency components of the average flux signal are the same as that of the measured time series.

**TABLE 2:** The coefficients of determination (R^2^) of mean wavelet analysis data between reconstructed signal, average flux signal, and measured time series. (a) All mean wavelet analysis data. (b)mean wavelet analysis data of frequency below 0.25 Hz.

R Jones (1852)	Subject1	Subject2	Subject3	Subject4
Reconstructed signal	0.990513	0.999836	0.967729	0.999832
Average flux signal	0.991526	0.999997	0.999914	0.999974

R Jones (1852)	Subject1	Subject2	Subject3	Subject4
Reconstructed signal	0.997176	0.999991	0.999912	0.999917
Average flux signal	0.999524	0.999997	0.999994	0.999973

#### 3.1.2 Numerical Results

**FIGURE 5:**
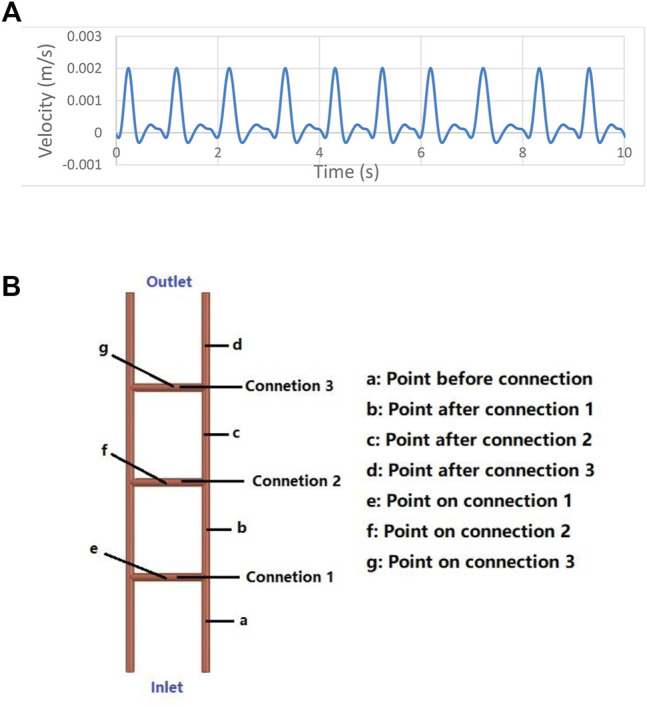
**(A)** Numerical input with a 5 s period. **(B)** Monitor points on the three-connected-tubes model.

In order to verify the influence of heart period variation on blood flow oscillation, the inlet velocity was set as below. The inlet velocity signal in the radial artery was based on the actual velocity waveform (Mills et al., 1970). The main period of the velocity signal was 5 s, including five different input heart periods, i.e., 0.93 s, 1 s, 1.14 s, 1 s, and 0.93 s. The velocity profile in the two main periods is shown in Figure 5A, and the maximum velocity is 2 mm/s.

**FIGURE 6:**
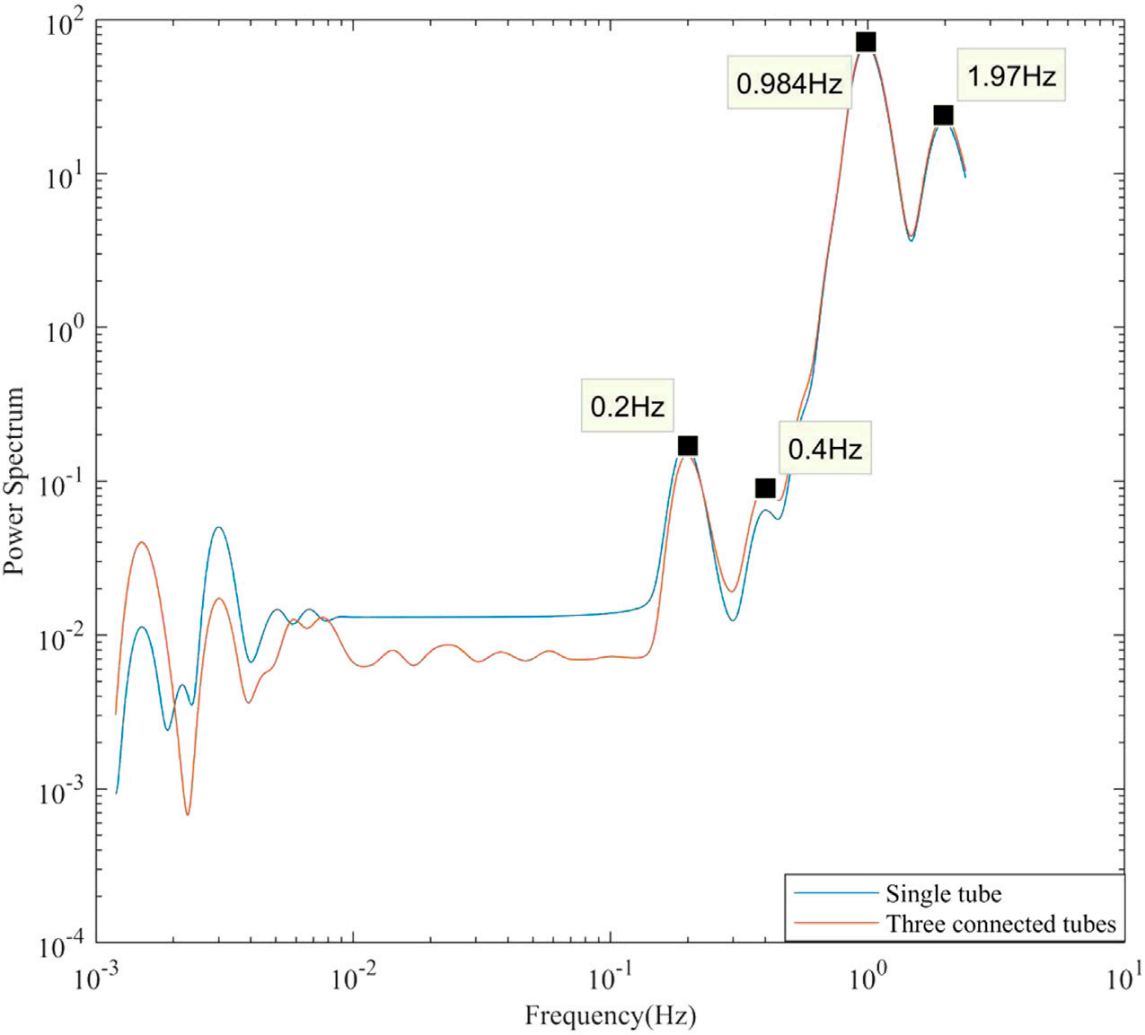
Single tube model and three-connected-tubes model (point before connection) with 0.2 Hz input frequency, mean wavelet analysis.

The mean wavelet analysis, which calculates the time-averaged power spectra from wavelet analyses, was performed to observe oscillation frequencies more clearly. Figure 6 shows the mean wavelet results for the three-connected-tubes model and the single tube model. The blue curve shows the results for the single tube model. 0.984 Hz is the mean input heart rate, 0.2 Hz is the input frequency, 0.4 and 1.97 Hz are the harmonic frequency of 0.2 and 0.984 Hz, respectively. The red curve shows the results for the three-connected-tubes model. The input frequencies (0.2 and ∼1 Hz) and several low frequencies components (0.01–0.1 Hz) are found at the point before connection, point a, in Figure 5B. The oscillation of blood flow in the three-connected-tubes model has several low frequencies compared to that in the single tube model. Thus, low frequencies in simulation results of the three-connected-tubes model are not only induced by the variation of heart period but also caused by the nonlinear vasculature.

**FIGURE 7:**
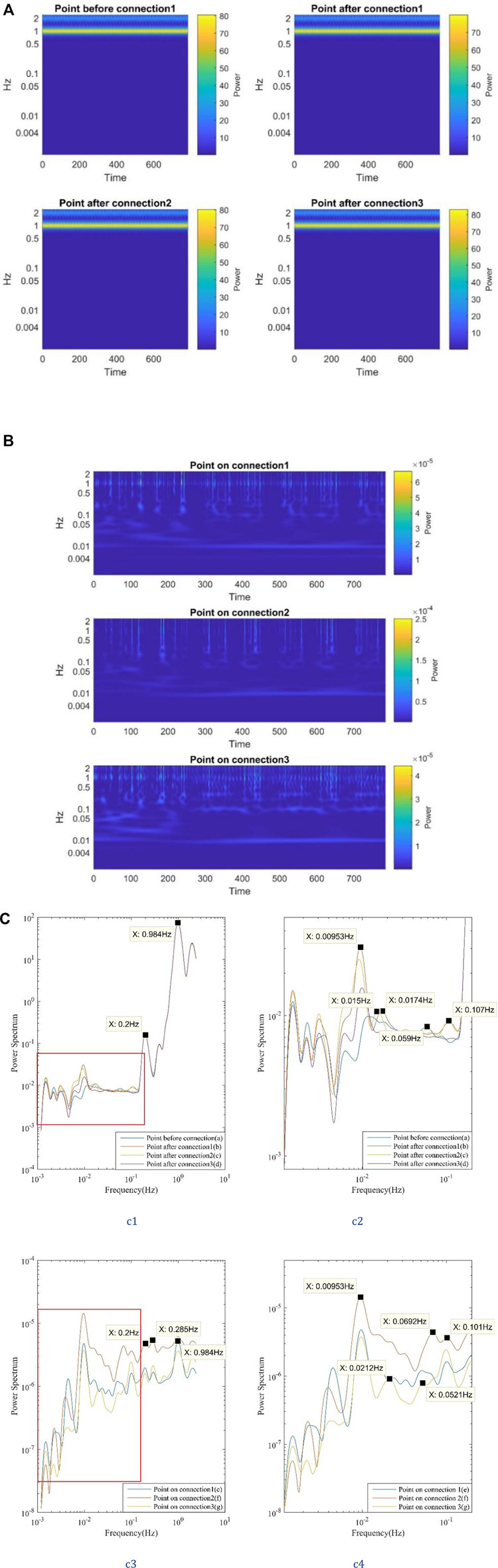
The wavelet analysis and the mean wavelet analysis of different monitor points on the three-connected-tubes model (2mm, 2 mm/s), with 0.2 Hz input frequency. **(A)** Three connected tubes model (2 mm, 2 mm/s), with 0.2Hz input frequency, wavelet analysis (points a, b, c and d). **(B)** Three-connected-tubes model (2 mm, 2 mm/s), with 0.2 Hz input frequency, wavelet analysis (points e, f and g). **(C)** Three-connected-tubes model (2 mm, 2 mm/s), with 0.2 Hz input frequency, mean wavelet analysis.

Data from other monitors shown in Figure 5B are analyzed to further investigate low-frequency oscillations at various locations on the three-connected-tubes model. Figure 7A displays the results for the four points (a, b, c, and d) on the straight tube. Figure 7B illustrates the results for the three monitors (e, f, and g) on the three connections. In Figure 7A the power mainly concentrates on ∼1 Hz in the wavelet analysis results of the points on the straight tube. In Figure 7B the same pattern is recognized for the three points on connections that the power spectrum is not purely concentrated on ∼1 Hz but spreads in the region from 0.5 to 2 Hz. Meanwhile, at these points on connections, the power spectrum of low-frequency oscillations varies with time which resembles the frequency feature discovered in the experiment data in Figure 5B.

Linear regression analysis helps to find the relationship between the inlet velocity and the computed velocity of the points on the connections (e, f, and g) separately. The coefficients of determination, R (Jones, 1852), are 0.007759, 0.002389, and 0.004832, respectively, which are close to zero, indicating that the correlation between the inlet velocity and the computed velocity of the points on the connections are nonlinear. By comparison, the linear regression of input velocity and the computed velocity of a point on the single tube generates an R^2^ of 0.992135 which is close to one and means a linear correlation. As a summary, the linear regression analysis results of the inlet velocity and computed velocity in the three-connected-tubes model show that the interconnected vasculature induces a nonlinear effect in the flow velocity at the points on the connections.

Mean wavelet analysis is performed for clearer observation of oscillation frequencies for the three-connected-tubes model, and the results are shown in Figure 7C. Curves within the red box in the left graphs, Figure 7C-c1 and Figure 7C-c3, are magnified and displayed in the right graphs, Figure 7C-c2 and Figure 7C-c4, respectively. As shown in Figure 7C-c1, at the points on the straight tube before and after connections, the inlet frequencies (0.2 and 1 Hz) play a dominant role in flow oscillations. Meanwhile, in the results on the connections shown in Figure 7C-c3, the vasomotion-related frequencies (∼0.1 Hz) have a higher power spectrum than that of inlet frequencies. In Figure 7C-c2 and Figure 7C-c4 several low frequencies oscillations are identified, i.e., 0.00953, 0.052, and 0.101 Hz. The 0.101 Hz frequency might be the subharmonic of 0.2 Hz input frequency. The power spectrum of 0.00953 Hz for the monitor on connection two is much higher than those on connections one and three.

The main straight tubes and the connections in the model are compared to arteries and branches in the vascular system, respectively. The findings above comply with the two facts below: (1) the vasomotion is mainly observed in microvasculature (Porret et al., 1995; Schmiedel et al., 2007; de Boer et al., 2014; Shaffer and Ginsberg, 2017); and (2) the power spectrum of 0.001–0.1 Hz may be higher than that at heart rate (Mayhew et al., 1996; Newman et al., 2009).

**FIGURE 8:**
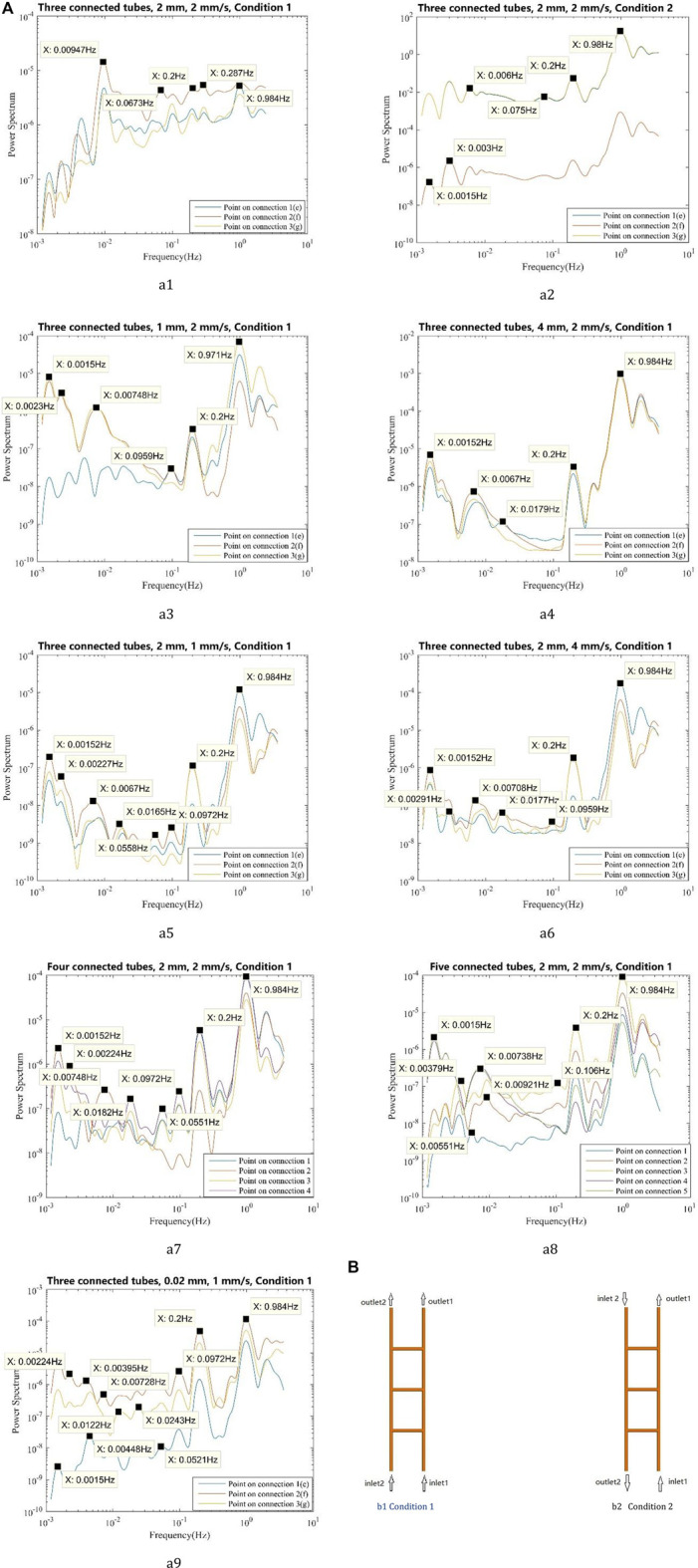
The wavelet analysis and the mean wavelet analysis of different models, with 0.2 Hz input frequency. **(A)** 0.2 Hz input frequency, different models with different diameters or with different maximum velocities or under different inlet and outlet conditions, wavelet analysis (points on the connections): **(a1)** Three-connected-tubes model, 0.2 Hz input frequency, 2 mm, 2 mm/s, Condition 1; **(a2)** Three-connected-tubes model, 0.2 Hz input frequency, 2 mm, 2 mm/s, Condition 2; **(a3)** Three-connected-tubes model, 0.2 Hz input frequency, 1 mm, 2 mm/s, Condition 1; **(a4)** Three-connected-tubes model, 0.2 Hz input frequency, 4 mm, 2 mm/s, Condition 1; **(a5)** Three-connected-tubes model, 0.2 Hz input frequency, 2 mm, 1 mm/s, Condition 1; **(a6)** Three-connected-tubes model, 0.2 Hz input frequency, 2 mm, 4 mm/s, Condition 1; **(a7)** Four-connected-tubes model, 0.2 Hz input frequency, 2 mm, 2 mm/s, Condition 1; **(a8)** Five -connected-tubes model, 0.2 Hz input frequency, 2 mm, 2 mm/s, Condition 1; **(a9)** Three-connected-tubes model, 0.2 Hz input frequency, 0.02 mm, 1 mm/s, Condition 1. **(B)** Different open-close states

The tubes of 1 and 4 mm diameter are simulated to investigate the influence of the tube diameter on frequencies. And the maximum inlet velocities of 1 mm/s and 4 mm/s are simulated to investigate the influence of velocity on frequencies. The number of connections increases to four and five to investigate the influence of geometry on frequencies. All the numerical results show a similar phenomenon that the vasomotion-related frequencies are prominent at the points on the connections compared to that at the points on the main tubes. The mean wavelet analysis results are shown in Figure 8A. From Figure 8A-a1, Figure 8A-a3, and Figure 8A-a4, the model with 2 mm diameter tubes has the most complex low-frequency components. From Figure 8A-a1, Figure 8A-a5, and Figure 8A-a6, using a maximum inlet velocity of 4 mm/s will induce less complex low-frequency components compared with using 1 mm/s and 2 mm/s velocities. In all these five cases, power spectrum of low-frequency components at points on connection two is higher than those at points on connection 1. In Figure 8A-a1, Figure 8A-a7, and Figure 8A-a8, the number of connected tubes changes from 3 to 5, and several low-frequency oscillations are identified in all these three models. The Womersley number is used to describe the frequency of pulsating flow associated with viscous effects. It is calculated by $\alpha =\frac{D}{2}\sqrt{\frac{2\pi f}{\gamma }}$ , where α is the Womersley number, D is the vessel diameter, f is the frequency, and γ is the dynamic viscosity of blood. The Womersley numbers for all these eight models range from 0.5 to 2, which correspond to the artery. The three-connected-tubes model with a 2 mm diameter is scaled to 1/100, and the diameter of the tubes in the scaled model is 0.02 mm. This model simulates the arteriole condition whose Womersley number is about 0.01. Figure 8A-a9 shows the mean wavelet result for the scaled model with 0.02 mm diameter tubes and 1 mm/s velocity. Several low-frequency oscillations are pointed out, and there is no obvious relationship between these low-frequency components and the variation in geometry.

Besides, different open-close states of the three-connected-tubes model are studied. The inlet and outlet conditions are shown in Figure 8B-b1 and Figure 8B-b2, and the mean wavelet results are shown in Figure 8A-a1 and Figure 8A-a2. Under condition 2, low-frequency components, such as 0.075 Hz, are induced and the ∼1 Hz frequency still plays a dominant role at monitors on the connected tubes. Different from the results under condition 1, the power spectrum of the point on connection two is much lower than that of the point on connections one and three.

## 4 Stochastic Resonance

### 4.1 HHT Results

**FIGURE 9:**
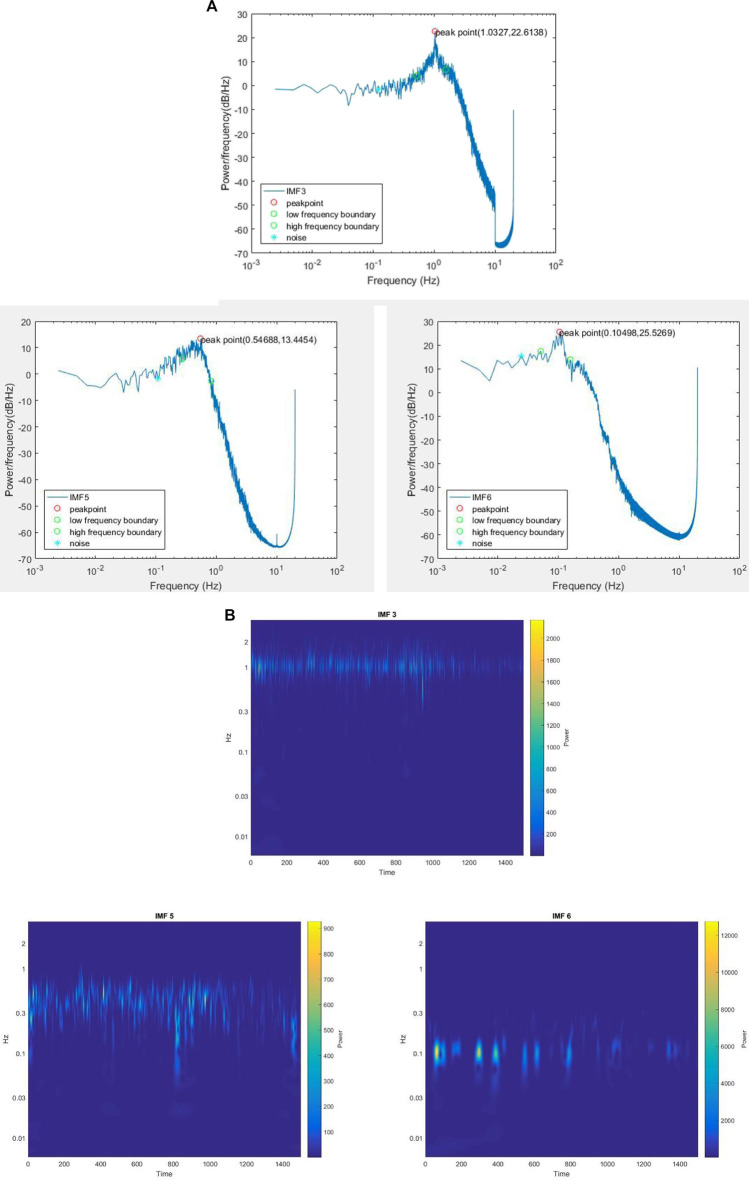
**(A)** The HHT IMFs and **(B)** the wavelet analysis for IMFs of measured data of radial artery at the wrist without adding noise.

Figure 9A is some IMFs decomposed from a group of LDF signal data without adding noise. The subsequent wavelet analysis for each IMF is shown in Figure 9B. The frequency band around 1 Hz is extracted in IMF 3, the frequency band around 0.3 Hz is extracted in IMF 5, and the frequency band around 0.1 Hz is extracted in IMF 6. The latter study is mainly focused on the frequency bands around 1 Hz, 0.3 Hz, and 0.1 Hz.

**FIGURE 10:**
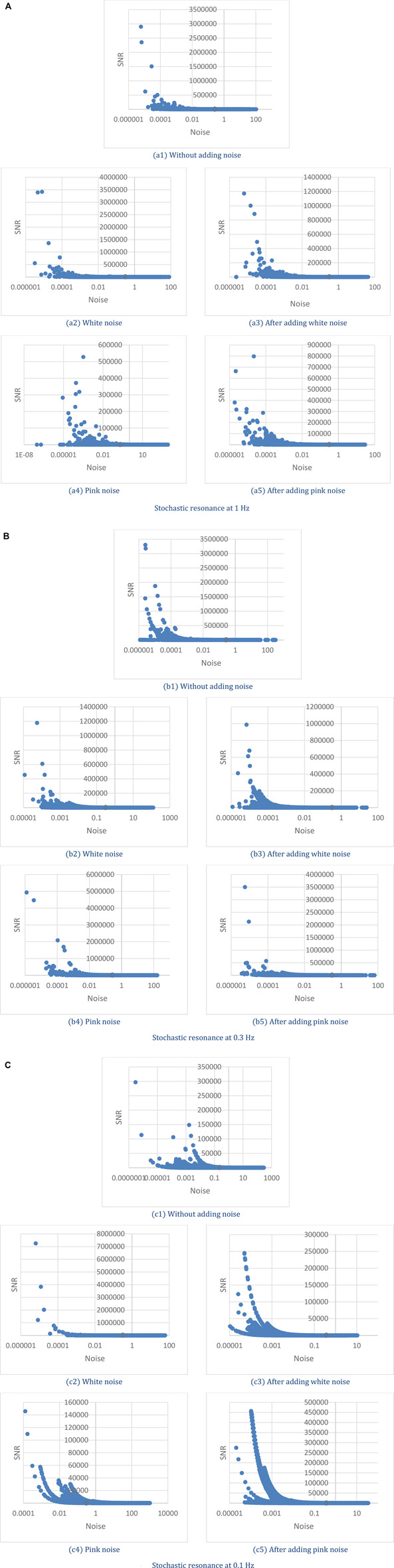
Stochastic resonance at **(A)** 1 Hz, **(B)** 0.3 Hz, and **(C)** 0.1 Hz.

The instantaneous SNR versus instantaneous noise intensity for frequency bands 1 Hz, 0.3 Hz, and 0.1 Hz are illustrated in Figures 10A, B, C, respectively. In certain noise intensity, the SNR ascends sharply to a peak value then decreases moderately with the continuous increase of noise intensity. There is inherent background noise in the human cardiovascular system (Yamamoto and Hughson, 1994), and regardless of whether noise is added, the inherent background noise of the human body is changing with time. The phenomenon of SR is observed in all these three frequency bands, which means the phenomenon of SR exists in the human cardiovascular system. Roughly from the figures, we cannot find the influence of adding the disturbance of noise to the pulse signal, the specific value of instantaneous SNR, and instantaneous noise intensity is then analyzed.

### 4.2 SNR for Different Disturbances of Noise

**FIGURE 11:**
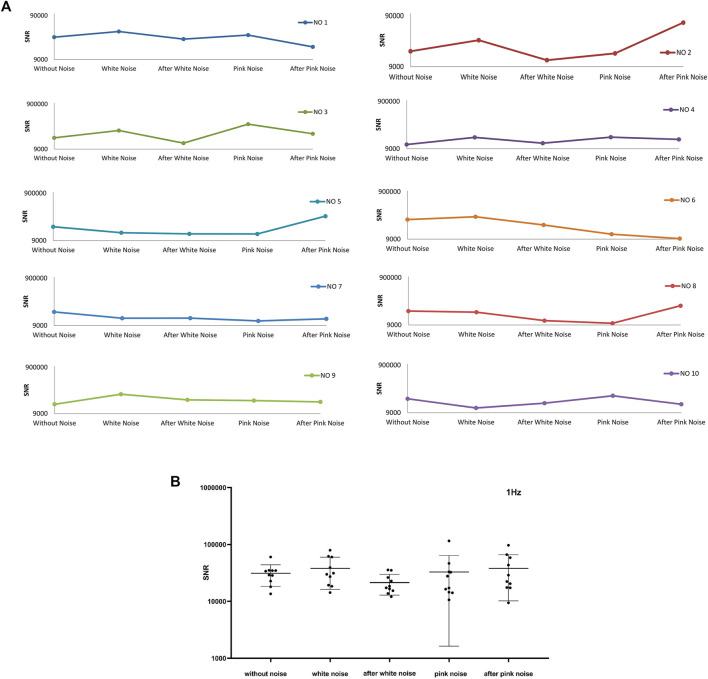
The average top 1000 SNR for frequency band around 1 Hz of ten experiments. **(A)** The average top 1000 SNR of ten experiments. **(B)** The total average top 1000 SNR of ten experiments and their mean ± SD at different noise conditions

From Figure 10, the maximum SNR is about 10^3^ to 10^6^ multiples of average SNR. Because higher instantaneous SNR contributes a major part, the entire SNR of a signal is represented by the average SNR of the top 1,000 largest instantaneous SNR. Figure 11A illustrates the average SNR of the top 1,000 largest instantaneous SNR of frequency band around 1 Hz. Six experiments represent similar responses to white noise, whose SNR raises after adding white noise and then falls after adding pink noise. And there is no commonality in SNR changing pattern which might be induced by pink noise. In Figure 11B, the blue line is flat, which indicates that there is little change in the value of the average SNR of the ten experiments for different noise conditions. The conclusion could be dropped that adding noise has little influence on the SNR of frequency around 1 Hz.

**FIGURE 12:**
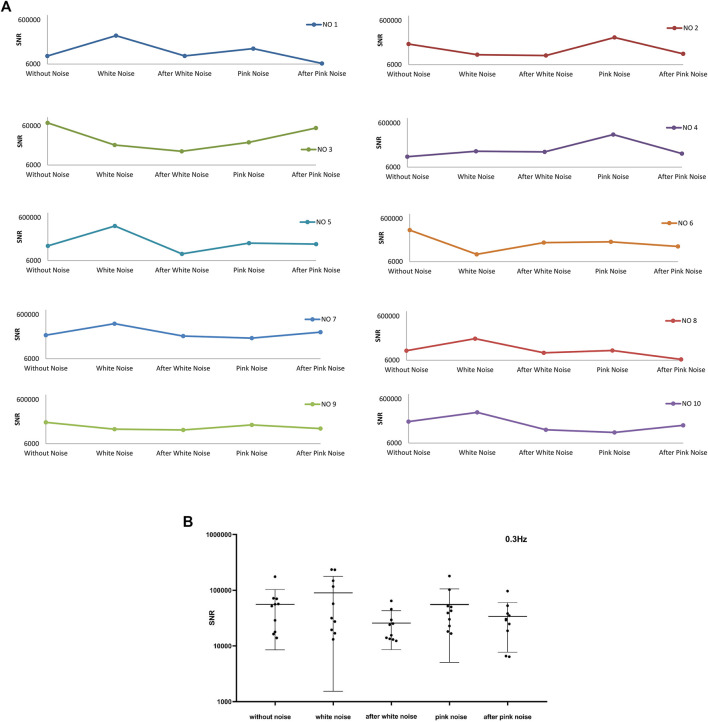
The average top 1000 SNR for frequency band around 0.3 Hz of ten experiments. **(A)** The average top 1000 SNR of ten experiments, **(B)** The total average top 1000 SNR of ten experiments and their mean ± SD at different noise conditions

Figure 12A shows the average SNR for the top 1,000 largest instantaneous SNR of frequency band around 0.3 Hz. There are six experiments’ SNR sharing the same trends that SNR heightens after adding white noise and reduces after stopping white noise. Eight experiments’ SNR strengthens after adding pink noise. Six of the eight experiments’ SNR reduce after stopping of pink noise, and two of the eight experiments’ SNR increase after withdrawing of pink noise. Combined with Figure 12B, both white and pink noise have impacts on the pulse signal in the frequency band around 0.3 Hz, and it is evident that after adding white noise the SNR will increase. This frequency band around 0.3 Hz relates to respiration activities. This result is consistent with the baroreflex system mentioned previously, and the baroreceptors detect the blood pressure ascent or descent in respiration activities (Lehrer and Vaschillo, 2008; Karemaker, 2009) and induce a threshold-like behavior related to SR^115,116^. Furthermore, the white noise will significantly enhance the frequency signals around 0.3 Hz, and the pink noise will moderate enhance the frequency signals around 0.3 Hz.

**FIGURE 13:**
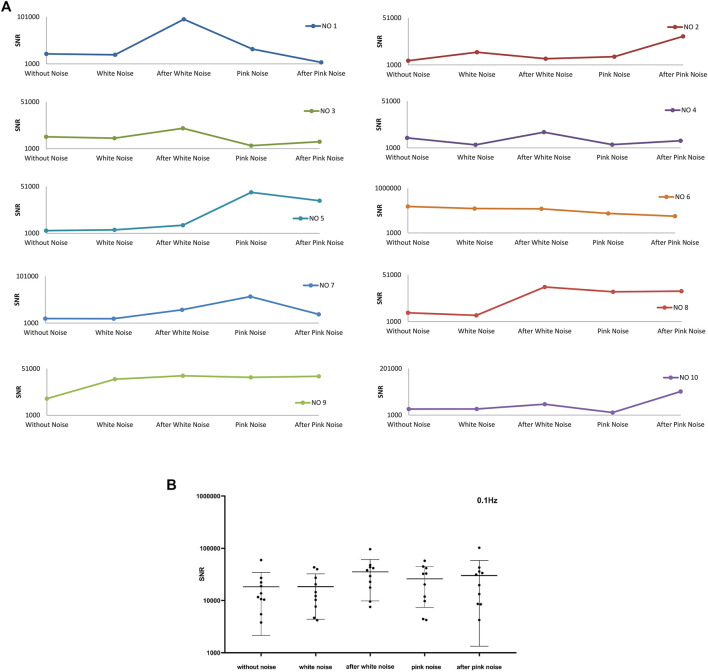
The average top 1000 SNR for frequency band around 0.1 Hz of ten experiments. **(A)** The average top 1000 SNR of ten experiments, **(B)** The average top 1000 SNR of ten experiments and their mean ± SD at different noise conditions.

Figure 13A shows the average SNR for the top 1,000 largest instantaneous SNR of frequency band around 0.1 Hz. There is a trend that almost all the ten experiments’ SNR will increase after withdrawing white noise. Same as the SNR change trend of the frequency bands around 1 Hz, the SNR has no apparent change in the pattern caused by adding pink noise. From Figure 13B, the SNR of frequency band around 0.1 Hz in the experiment condition after stopping white noise shows a clear difference to that before adding white noise. Therefore, it is evident that adding white noise then stopping it would induce the increase in SNR of frequency band around 0.1 Hz.

## 5 Conclusion

In this study, we carried out both experimental and numerical analyses to investigate the mechanism of vasomotion. The spectral analyses of the results lead to the following conclusions:

1) Based on the LDF measured radial artery blood flow signals at the wrist, two groups of data, reconstructed and average data, were acquired by constructing a sinusoidal flow velocity model using important features from the experimental data. The reconstructed and the experimental data displayed remarkably similar features in their results of wavelet analysis and mean wavelet analysis. More importantly, in the mean wavelet analysis plots, the experimental data, the reconstructed data, and the average data shared an almost identical low-frequency band, from 0.01 to 0.2 Hz. The physical meaning of average data is the average blood flux in each heart period, it can be speculated that the low-frequency band (0.01–0.2 Hz) in the experimental data originates from the variation of the heartbeat period, or the origin of vasomotion is from the heart beating period variation.
2) In numerical simulations, a single tube and a three-connected-tubes model with varying input velocity frequency were studied for the influence of the nonlinear vasculature on blood flow oscillations. The heartbeat and respiration are the only external excitations to the vascular system. The numerical results were processed at a variation of heart period with 0.2 Hz, and results show that several low-frequency blood flow oscillations are induced in three-connected-tubes model, indicating that the nonlinear vasculature would induce the low-frequency blood flow oscillations (0.01–0.1 Hz) which are related to vasomotion.
3) To investigate the effect of noise on the stochastic resonance of blood flow oscillation, the different noise interferences were added and the measured signals are decomposed by HHT. By comparing the changes of SNR for different frequency bands of different noise conditions, we could speculate that the noise has little influence on the frequency band related to the cardiac activities (∼1 Hz); both white and pink noise has impacts on the pulse signal for the frequency band related to the respiratory activities (∼0.3 Hz); adding white noise and then stopping would induce an SNR increase for the frequency band related to vasomotion (∼0.1 Hz).

## Data Availability

The original contributions presented in the study are included in the article/Supplementary Material, further inquiries can be directed to the corresponding author.
